# The Influence of Flightless I on Toll-Like-Receptor-Mediated Inflammation in a Murine Model of Diabetic Wound Healing

**DOI:** 10.1155/2013/389792

**Published:** 2013-02-18

**Authors:** Nadira Ruzehaji, Stuart J. Mills, Elizabeth Melville, Ruth Arkell, Robert Fitridge, Allison J. Cowin

**Affiliations:** ^1^Wound Healing Laboratory, Women's & Children's Health Research Institute, 72 King William Road, North Adelaide, Adelaide, SA 5006, Australia; ^2^Faculty of Health Sciences, The University of Adelaide, Adelaide, SA 5005, Australia; ^3^Early Mammalian Development Laboratory, Research School of Biological Sciences, Australian National University, Canberra, ACT 2601, Australia

## Abstract

Impaired wound healing and ulceration represent a serious complication of both type 1 and type 2 diabetes. Cytoskeletal protein Flightless I (Flii) is an important inhibitor of wound repair, and reduced Flii gene expression in fibroblasts increased migration, proliferation, and adhesion. As such it has the ability to influence all phases of wound healing including inflammation, remodelling and angiogenesis. Flii has the potential to modulate inflammation through its interaction with MyD88 which it an adaptor protein for TLR4. To assess the effect of Flii on the inflammatory response of diabetic wounds, we used a murine model of streptozocin-induced diabetes and Flii genetic mice. Increased levels of Flii were detected in Flii transgenic murine wounds resulting in impaired healing which was exacerbated when diabetes was induced. When Flii levels were reduced in diabetic wounds of Flii-deficient mice, healing was improved and decreased levels of TLR4 were observed. In contrast, increasing the level of Flii in diabetic mouse wounds led to increased TLR4 and NF-**κ**B production. Treatment of murine diabetic wounds with neutralising antibodies to Flii led to an improvement in healing with decreased expression of TLR4. Decreasing the level of Flii in diabetic wounds may therefore reduce the inflammatory response and improve healing.

## 1. Introduction

Up to 25% of people with diabetes can expect to develop a foot ulcer at some point in their lives [1]. Due to poor outcomes of existing therapies, lower extremity amputation is a common complication, affecting 15% of diabetics with foot ulcers, with one major amputation occurring every 30 seconds worldwide and over 2500 limbs lost a day [2]. The effectiveness of current treatments for diabetic foot ulcers is limited, and many patients with chronic, unhealed wounds need continual care. Understanding the processes involved in impaired wound healing will help to develop new therapeutic targets and tools for improving wound repair. 

One area of research which has been shown to be integral to the wound repair process is that of the actin cytoskeleton which is a filamentous network found in all cells and facilitates processes such as cellular adhesion, migration and contraction [3, 4]. One member of the actin cytoskeleton is Flightless I (Flii) which is a member of the gelsolin family of actin remodelling proteins [5]. Flii colocalizes with actin and microtubule-based structures, is required for normal actin distribution, and possesses Ca^2+^-independent G-actin binding activity as well as F-actin binding and severing activities [3, 6–8]. In addition to its role as a regulator of the cytoskeleton, the LRR domain allows Flii to bind a number of other proteins unrelated to actin including LRR Flii interacting proteins 1 and 2 (LLRFIP1 and LRRFIP2) and the double-stranded RNA binding protein TRIP [4, 9]. Flii is involved in numerous cellular activities including regulating transcription via coactivation of nuclear hormone receptors [10, 11] and regulation of beta-catenin-dependent transcription [12], important signalling pathways including the TLR pathway [13, 14], cellular polarity, asymmetric cell division [15], proliferation via interactions with calmodulin-dependent protein kinase type II [16], and inflammation and cytokine production via caspase activation and IL-1*β* maturation [17]. Flii-deficient mice have improved reepithelialisation after wounding while Flii overexpressing mice have impaired healing with larger wounds with reduced contraction, cellular proliferation and delayed reepithelialisation [18].

Increased inflammation is an important contributing factor in the failure to heal of diabetic foot ulcers [19]. Inflammation is an integral part of the wound healing process and is regulated by toll-like receptors (TLRs). TLRs are key innate immune receptors that alert the immune system to tissue damage and mediate the inflammatory response. The human TLR family consists of 10 members structurally characterised by the presence of a leucine-rich repeat (LRR) domain in their extracellular domain and toll/interleukin-(IL-) 1 receptor (TIR) domain, in their intracellular domain. Through their intracellular TIR domain TLRs activate or deactivate signalling pathways that generate cytokine and chemokine production and thereby regulate inflammatory responses. TLR signalling is tightly regulated to control the intensity and duration of inflammation [12]. TLRs are expressed on a wide variety of cells including macrophages and neutrophils, and they respond to an array of viral, bacterial, and fungal ligands as well as cellular debris [13]. The receptors convey their specificity through the utilization of different adaptor proteins such as myeloid differentiation factor 88 (MyD88), TIR-associated protein (TIRAP), TIR domain containing adaptor protein-inducing IFN-*β* (TRIF), and TRIF-related adaptor molecule (TRAM) [13–15]. Upon activation, the adaptor proteins promote signalling to result in the expression of proinflammatory cytokines, growth factors, and interferons. MyD88 has been shown to play an important role during wound healing as MyD88 knockout mice have impaired wound healing [16] with wounds showing reduced contraction, decreased and delayed granulation tissue formation, and reduced blood vessel density [16]. 

The LRR region of Flii shares 29% sequence identity and 42% similarity to TLR4 [20]. Through its interaction with MyD88, it has been suggested that Flii can modulate inflammation by suppressing TLR4-MyD88-mediated activation of NF-*κ*B [21]. Conversely, a reduction in the Flii level may enhance activation of NF-*κ*B and increase cytokine secretion [20]. Several studies investigating the effect of Flii on TLR signalling in murine macrophages suggest that Flii can sequester activator proteins such as LPS and adaptor proteins such as MyD88 resulting in reduced cytokine expression [10, 11, 16, 17]. In this study we used mice with low (Flii^+/−^), normal (WT), and high (Flii^Tg/Tg^) Flii gene expression levels to investigate the function of Flii in a murine wound healing model of streptozocin-induced type 1 diabetes. We also investigated whether modulation of Flii by exogenous application of Flii neutralising antibodies improved diabetic wound healing via effects on TLR-mediated inflammation. 

## 2. Materials and Methods

### 2.1. Antibodies

Mouse monoclonal anti-Flii antibodies raised to the LRR domain of the human Flii protein and the rabbit anti-human MyD88 antibody were obtained from Santa Cruz Biotechnology (VIC, Australia). Rabbit anti-human TLR4 and TLR9 antibodies were obtained from Imgenex (SA, Australia), mouse anti-human CD14 antibody and mouse anti-human CD16 antibody from BD Biosciences (NSW, Australia), and rabbit anti-human NF-*κ*B antibody from Abcam (NSW, Australia). All antibodies were used at a 1 : 100 dilution. The appropriate secondary antibodies were used depending on the fluorescence required—goat anti-mouse Alexa Fluor 488 and goat anti-mouse Alexa Fluor 594 were obtained from Life Technologies (VIC, Australia) and also used at a 1 : 100 dilution. Flii is a highly conserved protein with 95% homology between mice and humans [19]. The Flii neutralising antibodies (FnAbs) used in *in vivo *diabetic mouse trials were mouse monoclonal anti-Flii antibodies raised against the N-terminus of the LRR domain of the human Flii protein [22] and obtained from Monoclonal Antibody SA Technologies (SA, Australia). Mouse IgG antibody used as a control *in vivo* diabetic mouse was obtained from Sigma (MO, USA) 

### 2.2. Animal Studies

All experiments were approved by the Women's and Children's Health Network Animal Ethics Committee following the Australian Code of Practice for the Care and the Use of Animals for Scientific Purposes. Studies were performed using mice with a BALB/c background. Three strains of mice were used in this study with low (Flii^+/−^), normal (WT), and high (Flii^Tg/Tg^) Flii gene expression levels. Mice lacking one copy of the Flii gene, the double knockout being embryonically lethal [23], were made as described previously [23] and will be written as Flii^+/−^. Transgenic mice carrying two extra copies of human Flii gene incorporated into mouse genome will be written as Flii^Tg/Tg^. 

### 2.3. Murine Model of Diabetic Wound Healing

Six female Flii^+/−^, WT, and Flii^Tg/Tg^ mice of 12–16 weeks of age and weighing 20–35 g were used for the induction of diabetes. Streptozocin (STZ) was used to induce type 1 diabetes (Sigma-Aldrich, MO, USA). STZ is toxic to the pancreatic beta-islet cells, rendering the mouse unable to produce adequate amount of insulin. Mice were given one intraperitoneal (IP) injection of STZ for 5 consecutive days (STZ: 50 mg/kg) in citrate buffer of pH 6.5. This dose was chosen based on previously reported studies [24]. Age-matched nondiabetic control animals were treated with an equivalent dose of vehicle (citrate buffer alone). Diabetic symptoms were observed closely, and nonfasting blood glucose levels (BGLs) were tested weekly by tail vein sampling. To maintain body weight and prevent ketoacidosis, animals with confirmed diabetes were maintained with subcutaneous injection of insulin (1 IU, Mixtard 30/70, Novo-Nordisk, NSW, Australia). Mice were tested for sufficient levels of hyperglycaemia at 6 weeks after the last STZ injection, and only those with blood glucose levels greater than 15 mmol/L were wounded. Diabetic animals were wounded using a 6 mm biopsy punch (Stiefel Laboratories, NSW, Australia). Anaesthesia was induced by inhalation of isoflurane (5% induction at 2 L/min and 2% maintenance at 500 mL/min). To expose the skin, hair was removed by shaving and then application of hair removal cream (Veet, Reckitt Benckiser, NSW, Australia). Two 6 mm full thickness wounds, one on each side of the midline, were created on the dorsum of the mouse. Temgesic (buprenorphine 0.05 mg/kg) was administered post-operatively to provide analgesia for up to 8 hours. The mice were euthanized at 7 days following wounding. Digital photographs of the wounds were taken at 0 and 7 days after wounding. A ruler was aligned next to the wound to allow direct wound area measurements to be made. Wounds were fixed in formalin and processed for histology and immunohistochemistry. 

An additional cohort of female WT diabetic mice were injected intradermally around the wound margins with 200 *μ*L of FnAb (50 *μ*g/mL; *n* = 10) or mouse IgG control (50 *μ*g/mL; *n* = 10) immediately after surgery and at 1 and 2 days after wounding. Digital photographs of wounds were taken at days 0 and 7 after wounding. All animals were euthanized at day 7 post-wounding with the wounds harvested, fixed in formalin, and processed for histological analysis. 

### 2.4. Histology, Immunohistochemistry, and Image Analysis

Histological sections (4 *μ*m) of mouse wounds were cut and stained with haematoxylin and eosin or subjected to immunohistochemistry following antigen retrieval as described previously [25]. Briefly, following antigen retrieval, sections were blocked in 3% normal goat serum, primary antibodies against Flii (1 : 100), TLR9 (1 : 100), TLR4 (1 : 100), MyD88 (1 : 100) and NF-*κ*B (1 : 100) were applied and incubated overnight at 4°C. Appropriate Alexa Fluor 488-conjugated secondary antibodies (1 : 100) were used and incubated for 1 hour. Fluorescence intensity was determined using AnalySIS software package (Soft Imaging System GmbH, Munster, Germany), and optical fluorescence in the epidermis and dermis of the wounds was analysed as previously described [25]. Negative controls included replacing primary antibodies with normal mouse or normal rabbit IgG. Primary or secondary antibodies were omitted to verify the staining and detect nonspecific binding. All control sections had negligible immunofluorescence. 

### 2.5. Histological Image Analysis

Histological image analysis was performed using the Digital Microscope Camera ProgRes C5 (JENOPTIK Laser, Jena, Germany). Wound size was determined by manually drawing below the clot or the portions of the wound that were not covered by epidermis. Dermal wound gape was determined by measuring between the dermal wound margins. Fluorescent images were taken using an Olympus IX81 (Olympus Australia, Melbourne, VIC, Australia) at a magnification of ×20. The fluorescent intensity of the staining was calculated using Image Pro-Plus software (Media Cybernetics, MD, USA).

### 2.6. Statistical Analysis

Statistical significance was calculated using a paired Students *t*-test or analysis of variance. A *P* value of 0.05 or less was considered significant.

## 3. Results

### 3.1. Diabetic Wounds Heal Faster in Mice with Low Levels of Flii Gene Expression

**Figure 1:**
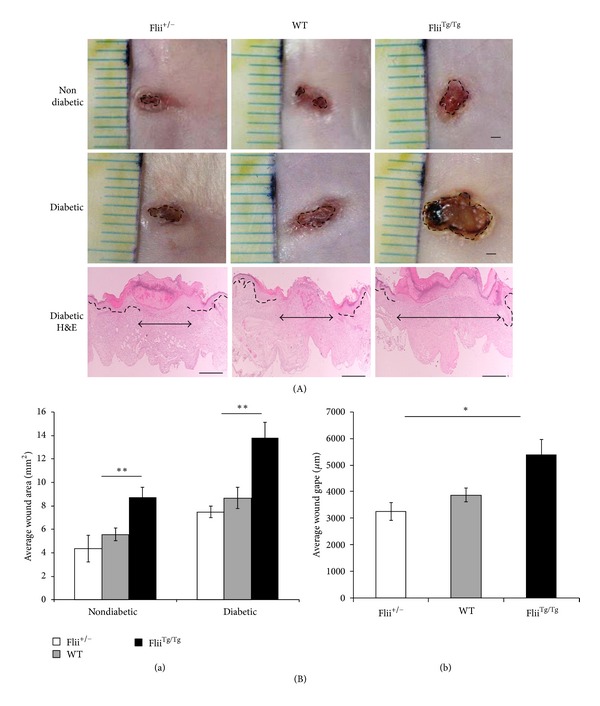
Flii delays wound healing in diabetic murine wounds after 7 days.(A) Macroscopic images of day 7 nondiabetic and diabetic mouse wounds in the Flii ^+/−^, WT, and Flii^Tg/Tg^ mice and representative histological H&E staining of day 7 diabetic wounds. Scale bars = 1 mm for macroscopic pictures, and for the histology magnification was ×4 and scale bars = 100 *μ*m. (B) shows graphical representations of the wound areas of the day 7 (a) wound areas of the three Flii genotype diabetic wounds (*n* = 6) and (b) the microscopic dermal wound gape of the day 7 diabetic wounds where **P ≤ 0.01 and *P ≤ 0.05 (*n* = 6).

To assess the biological function of Flii and determine the effect of Flii gene modulation on diabetic wound healing, three lines of mice were used expressing low (Flii^+/−^), normal (Flii^+/+^), and high (Flii^Tg/Tg^) levels of Flii. Representative digital images of wounds at 7 days subsequent to wounding are shown in Figure 1(A). Overexpression of Flii in diabetic and nondiabetic wounds resulted in delayed wound closure at day 7 following wounding (Figure 1(A)). In contrast, wound area was decreased significantly when Flii levels were reduced (Flii^+/−^; *P* = 0.01) compared to Flii^Tg/Tg^ mice at day 7 following wounding (Figure 1(B)(a)). Representative microscopic images of day 7 wounds are shown in Figure 1(A). Histological assessment of these diabetic mouse wounds showed that at day 7 the dermal wound gape was significantly smaller in Flii-deficient mice (Flii^+/−^) compared with Flii overexpressing (Flii^Tg/Tg^) mice (Figure 1(B)(b); *P* = 0.05). 

### 3.2. Treatment of Diabetic Wounds with Flii Neutralising Antibodies (FnAbs) Improves Wound Healing

**Figure 2:**
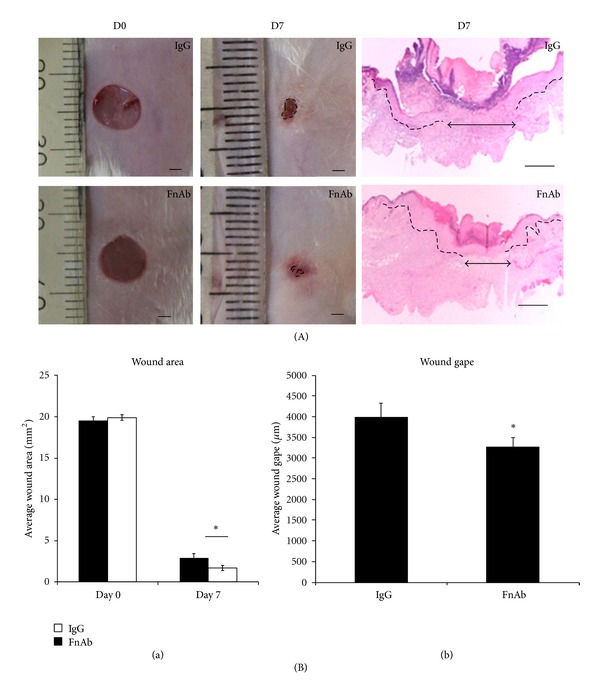
Healing can be improved by the application of Flii neutralizing antibodies. (A) shows macroscopic images of day 0 and day 7 excisional WT diabetic wounds treated with IgG isotype control (*n* = 10) and Flii neutralizing antibody (FnAb) (*n* = 10). Representative pictures of H&E staining of day 7 diabetic wounds treated with IgG and FnAb. (B) Graphical representation of the (a) wound areas and (b) histological wound gapes of the days 0 and 7 IgG control-treated and FnAb-treated WT diabetic wounds where *P ≤ 0.05 (*n* = 10).

Previous studies have shown that intradermal injection of Flii neutralising antibodies reduces the level of Flii in wounds and improves healing [26]. FnAbs were injected intradermally at days 0, 1, and 2, and representative images of macroscopic (at days 0 and 7) and microscopic (day 7) appearances of diabetic wounds are shown in Figure 2(A). Intradermal administration of FnAb to diabetic wounds resulted in a 1.9-fold decrease in average wound area (Figure 2 (B)(a)) and histological wound gape (Figure 2(B)(b)) compared to IgG-treated WT diabetic controls (Figure 2(B) (a) and (b); *P* ≤ 0.05; IgG versus FnAb). 

### 3.3. Elevating Flii Gene Expression Increases TLR4 in Diabetic Mouse Wounds

**Figure 3:**
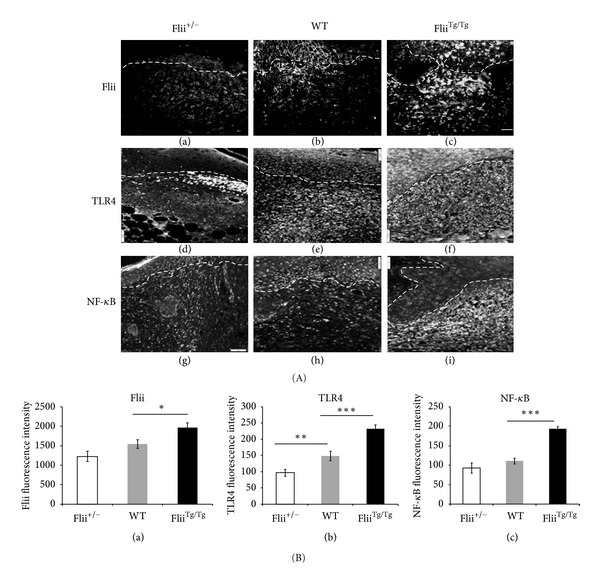
Concomitant increase in TLR4 and NF-*κ*B staining occurs with increasing levels of Flii. (A) Representative images for Flii (a)–(c), TLR4 (d)–(f), and NF-*κ*B (g)–(i) immunostaining of the three Flii genotypes (Flii^+/−^, WT, and Flii^Tg/Tg^). (B) Graphical representation of (a) Flii, (b) TLR4, and (c) NF-*κ*B in Flii^+/−^, WT, and Flii^Tg/Tg^ day 7 diabetic wounds. *P ≤ 0.05; **P ≤ 0.01; ***P ≤ 0.001 (*n* = 6) and scale bar = 100 *μ*m.

Immunofluorescence staining of day 7 diabetic wounds in Flii^+/−^, WT, and Flii^Tg/Tg^ mice shows increased Flii in Flii^Tg/Tg^ diabetic wounds > WT > Flii^+/−^ wounds (Figure 3(A)) with significantly less Flii staining being observed in day 7 diabetic Flii^+/−^ and WT wounds (*P* = 0.04; Flii^+/−^ versus Flii^Tg/Tg^; *P* = 0.05; WT versus Flii^Tg/Tg^; Figure 3(B)(a)). The presence of foreign molecules and pathogens was detected by a family of receptors known as toll-like receptors (TLRs), which contribute to prolonged inflammation [27]. To assess if altered levels of Flii affected TLR4 expression, diabetic wounds were stained for TLR4. Increasing Flii leads to a concomitant increase in TLR4 expression (Figure 3(A)(d)–(f)). Flii deficiency caused a significant reduction in TLR4 expression (Figure 3(B)(b)) at day 7 following wounding which was significantly lower than WT and Flii^Tg/Tg^ wounds (*P* ≤ 0.01 WT versus Flii^+/−^; *P* ≤ 0.001 WT versus Flii^Tg/Tg^). Given that the inflammatory response in diabetic wounds was associated with increased TLR4 expression, we next proceeded to test if the downstream molecule NF-*κ*B production was also affected. Figure 3(A) shows that NF-*κ*B expression was also increased in Flii overexpressing wounds (5-fold higher than WT diabetic wounds) (Figure 3(B)(c); *P* ≤ 0.001Flii^Tg/Tg^ versus WT). A reduction in NF-*κ*B was observed between Flii^+/−^ wounds and WT, but this was not statistically significant (Figure 3(B)(c)). 

### 3.4. Flii Neutralising Antibodies Reduce TLR4 Expression in Diabetic Wounds

**Figure 4:**
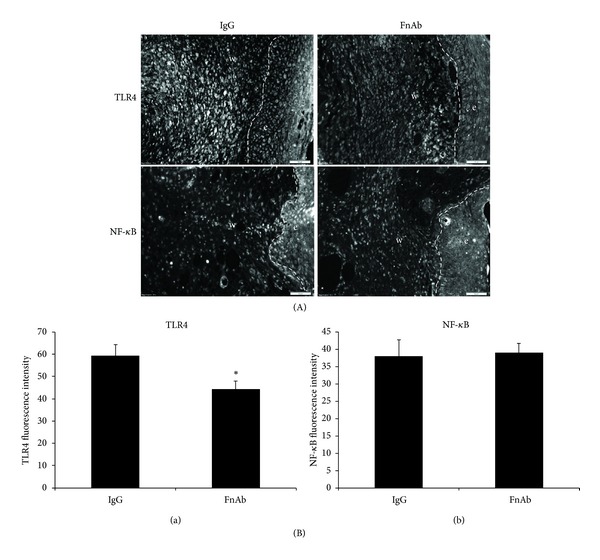
Modulation of Flii by exogenous application of Flii neutralising antibodies (FnAbs) reduces TLR4 expression in diabetic wounds. (A) Representative images of TLR4 and NF-*κ*B staining of day 7 diabetic wounds treated with IgG and FnAb. (B) Graphical representation of (a) TLR4 and (b) NF-*κ*B staining of day 7 diabetic wounds treated with IgG and FnAb. *P ≤ 0.05.

Given that intradermal application of FnAb resulted in improved healing of diabetic wounds (Figure 2(A)), we proceeded to test whether this was in part due to modulated levels of TLR4-mediated inflammation. TLR4 and NF-*κ*B expression was quantified, and representative images are shown in Figure 4(A). Treatment with FnAbs resulted in a significant decrease in TLR4 expression (Figure 4(B); *P* ≤ 0.05 IgG versus FnAb) whereas NF-*κ*B expression in day 7 FnAb-treated wounds remained unchanged compared to IgG controls (Figure 4(B)(b)). 

## 4. Discussion

Flii has been identified as a protein that can inhibit the rate of healing by reducing the migration of keratinocytes and fibroblasts and limiting the degree of wound contraction [18, 22, 28]. Flii deficiency is associated with improved reepithelialisation in acute wounds [18] while Flii overexpressing mice have impaired wound healing with delayed reepithelialisation. Here we have investigated if Flii is involved in the impaired healing associated with diabetic wounds. Wound healing was impaired as Flii levels increased, and this impairment was exacerbated when diabetes was induced. Flii is upregulated during the wound repair process [18, 25] and is constitutively secreted by two of the major cell types found in wounds: fibroblasts and macrophages in response to wounding both *in vitro* [29] and *in vivo *[22, 30]. Recent studies show that Flii is also secreted through a nonclassical late endocytic/lysosomal pathway of secretion by fibroblasts and macrophages [29]. Addition of Flii monoclonal neutralising antibodies as a means of reducing the Flii protein in the wound environment was able to counteract the negative effect of Flii on wound healing [18], and treatment of murine diabetic wounds with neutralising antibodies to Flii led to an improvement in healing suggesting that high levels of Flii in diabetic wounds contribute to wound chronicity. 

Inflammation is an essential component of the normal wound healing process; however excessive inflammation is detrimental to this process [31]. Disproportionate inflammation is one of the major contributing factors to the formation of diabetic ulcers as these chronic wounds often have an unregulated and excessive inflammatory reaction [31, 32]. The innate immune system detects foreign particles such as bacteria, fungi, and viruses via pathogen-associated molecular pattern (PAMP) molecules leading to the activation of the inflammatory response via toll-like receptors (TLRs) which recognise PAMPs [33, 34]. TLRs have also been linked to diabetes with studies showing that TLR-immune activation can result in activation of proinflammatory pathways leading to autoimmunity which may cause the onset of diabetes [27]. Previous reports suggested that stimulation of TLR4 leads to activation of the downstream transcriptional regulator NF-*κ*B resulting in cytokine secretion [21], and both TLR4 expression and pro-inflammatory cytokine NF-*κ*B were elevated in the murine diabetic wounds which would further contribute to inflammation and chronicity. In Flii-deficient diabetic mouse wounds, decreased TLR4 and NF-*κ*B were observed and when Flii neutralising antibodies were administered to diabetic wounds, a decrease in TLR4 expression was seen suggesting a dampening of the inflammatory response when Flii levels were reduced. Interestingly, the effect of FnAb was specific to TLR4 and did not appear to affect the production of the proinflammatory cytokine NF-*κ*B suggesting that alternative pathways may still be active in these wounds. 

*In vitro* studies have previously shown that the LRR region of Flii shares 29% sequence identity and 42% similarity to TLR4 [20] suggesting that Flii may influence TLR signalling. However, while *in vitro* studies showed that through its interaction with MyD88, Flii was able to negatively regulate the TLR4-MyD88-mediated activation of NF-*κ*B and the subsequent cytokine secretion in macrophages [20, 21], our studies show that *in vivo*, in mouse diabetic wounds the increased levels of Flii appear to correlate with an increase in the expression of TLR4 and its signalling protein NF-*κ*B. This is clearly in opposition to the findings in these cell-based studies but may be accounted for by the differing *in vivo* and *in vitro* environments. The time points investigated also differ with the *in vitro *studies looking at responses from 3 to 6 hours whereas this wounding study investigated time points which were measured in days rather than hours. It is, however, not inconceivable that Flii may have a dual role in wounding and in the inflammatory response which depends on the time point investigated. 

In conclusion, inflammation is an integral component of the normal wound healing process and occurs even in the absence of infection; however excessive and prolonged inflammation impairs healing. Flii is a multifunctional protein and is currently emerging as a regulator of inflammation; however, whether it is pro-or anti-inflammatory is still to be determined. Our *in vivo* studies show that reducing the expression of Flii in diabetic murine wounds improves healing and reduces the proinflammatory response. Being able to manipulate the level of inflammation in a wound would greatly improve the wound healing outcomes of patients with diabetes, and it remains to be elucidated whether neutralisation of Flii in human diabetic wounds could help improve healing of these chronic nonhealing ulcers.
